# Laparoscopic Nephrectomy, *Ex Vivo* Partial Nephrectomy, and Autotransplantation for the Treatment of Complex Renal Masses

**DOI:** 10.1155/2014/354104

**Published:** 2014-11-24

**Authors:** Jasmir Gopal Nayak, Joshua Koulack, Thomas Brian McGregor

**Affiliations:** ^1^Section of Urology, University of Manitoba, Z3013-409 Taché Avenue, Winnipeg, MB, Canada R2H 2A6; ^2^Department of Urology, University of Washington, BB1121-1959 NE Pacific Street, Seattle, WA 98195, USA; ^3^Section of Vascular Surgery, University of Manitoba, GF547-820 Sherbrook Street, Winnipeg, MB, Canada R3A 1R9

## Abstract

In the contemporary era of minimally invasive surgery, very few T1/T2 renal lesions are not amenable to nephron-sparing surgery. However, centrally located lesions continue to pose a clinical dilemma. We sought to describe our local experience with three cases of laparoscopic nephrectomy, *ex vivo* partial nephrectomy, and autotransplantation. Laparoscopic donor nephrectomy was performed followed by immediate renal cooling and perfusion with isotonic solution. Back-table partial nephrectomy, renorrhaphy, and autotransplantation were then performed. Mean warm ischemia (WIT) and cold ischemic times (CIT) were 2 and 39 minutes, respectively. Average blood loss was 267 mL. All patients preserved their renal function postoperatively. Final pathology confirmed pT1, clear cell renal cell carcinoma with negative margins in all. All are disease free at up to 39 months follow-up with stable renal function. In conclusion, the described approach remains a viable option for the treatment of complex renal masses preserving oncological control and renal function.

## 1. Introduction

Laparoscopic and robotic partial nephrectomy are the gold standard treatments for small renal masses [1, 2]. This is particularly important for patients with a solitary kidney or preexisting renal impairment. Nephron-sparing surgery minimizes the risk for further renal insufficiency or dialysis; the complications of which are well described. However, complex renal masses, typically involving a central or hilar location, require meticulous dissection and renorrhaphy that is difficult to achieve by standard* in situ* techniques. Initially described by the late Dr. Novick et al. [3, 4], nephrectomy followed by* ex vivo* partial nephrectomy and autotransplantation provides an alternative approach with many advantages. Surgical exposure is maximized by the* ex vivo* nature of the procedure and the bloodless field, allowing for meticulous dissection. In addition, the kidney can be cooled and flushed with preservative solution. This technique requires experience in renorrhaphy, vascular reconstruction, and renal transplantation. Laparoscopic donor nephrectomy and allotransplantation is a standard urological procedure at our institution. Based on this experience, similar techniques were applied to 3 patients who presented with complex renal masses within a solitary kidney or with significant preexisting renal impairment. We present our experience with three cases of RCC that were successfully treated with* ex vivo* partial nephrectomy and autotransplantation. All patients provided written informed consent with guarantees of confidentiality.

## 2. Case Presentation

**Figure 1:**
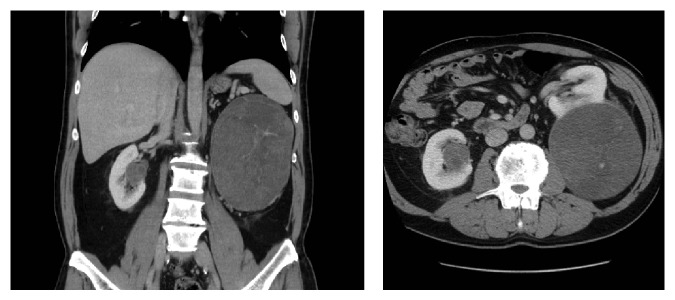
Infused axial and coronal CT abdominal images of patient 1.

**Figure 2:**
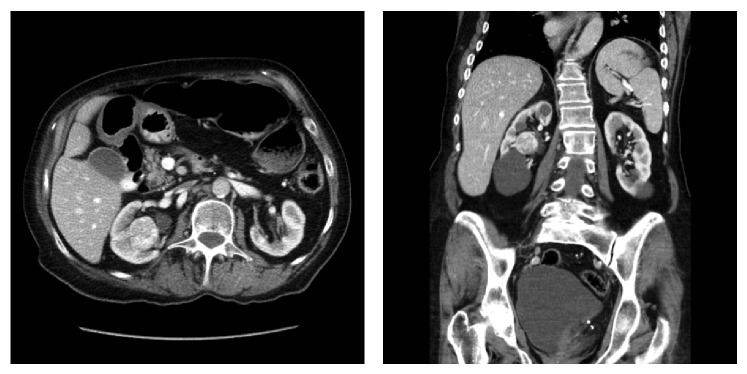
Axial and coronal infused CT imaging of patient 3.

Patient 1 was a 56-year-old male who was found to have bilateral renal masses with a 17 cm left renal mass and a 4 cm, centrally located right renal mass. The patient successfully underwent a left laparoscopic nephrectomy leaving him with a central renal mass in his solitary right kidney (Figure 1). Preoperative renal function demonstrated a serum creatinine of 116 *μ*mol/L; GFR 101.6 mL/min. Patient 2 is a 76-year-old female with a history of stage IV chronic kidney disease that presented with a centrally located mass in her solitary functioning kidney. Patient 3 is a 75-year-old female with significant comorbidities including stage IIIb chronic kidney disease that presented with an incidental centrally located renal mass (Figure 2). The R.E.N.A.L. (radius, exophytic/endophytic, nearness to collecting system or sinus, anterior/posterior, and location relative to polar lines) nephrotomy scores [5] were 10 h, 11 h, and 10 h for patients 1–3, respectively.

All patients were positioned lateral decubitus, followed by Hassan entry and abdominal insufflation. The colon was mobilized medially and the renal artery and vein are dissected proximally to provide sufficient length for autotransplantation. The ureter was dissected distally to the common iliac artery. Once the kidney had been completely mobilized, the artery and vein were divided. The specimen was extracted through a Gibson incision. The renal unit was immediately placed in an ice-bath solution and flushed with histidine-tryptophan-ketogluterate (HTK) solution. On the back table, perirenal fat was removed and segmental vessels were identified. Once the tumor had been dissected away from other vital structures, frozen sections were performed confirming negative resection bed margins. Collecting system defects were identified and repaired following retrograde instillation of methylene blue through the ureter. The resection defect was packed with absorbable hemostatic agents and the renal capsule was reapproximated using bolsters and absorbable sutures. The patients were subsequently repositioned supine and the Gibson incision was extended to allow for renal transplantation into the ipsilateral pelvis. The kidney was anastomosed end-to-side to the external iliac artery and vein. The ureteroneocystotomy was performed over a stent. A foley catheter and pelvic drain were placed.

For all cases, laparoscopic nephrectomy was performed without event. WIT's were negligible at less than 2 minutes. Working under cold ischemic conditions, the index renal lesions were meticulously dissected from the kidney with subsequent renorraphy. CIT's were 35, 40, and 42 minutes for patients 1–3, respectively. The renal autotransplantation was then carried out uneventfully in each case. Mean estimated blood loss was 267 mL (range: 100–600 mL). Mean operative time was 5.5 hours. Patient 1 developed urinoma postoperatively successfully managed with a percutaneous drain. Otherwise all patients recovered from surgery without complication. Length of stay ranged from 8 to 19 days. Final pathological evaluation demonstrated Furhman grade (FG) II pT1a, FG III pT1b, and FG II pT1a, clear cell RCC with clear margins in patients 1–3, respectively. After a median follow-up of 25.8 months (range: 18.9–39.3 months), all patients were disease free with stable renal function. The pre-/postsurgery GFR's were 103/80, 25/27, and 34/39 mL/min for patients 1–3, resulting in a mean change in serum creatinine of +13.7 *μ*mol/L and GFR of −5.1 mL/min.

## 3. Discussion

We present the largest series of this approach, using laparoscopic nephrectomy, with the longest followup in the literature, and have shown that complex renal masses can be managed through renal preserving methods while maintaining oncological control. Despite significant technological advances in minimally invasive surgery over the past 2 decades, complex renal masses continue to pose a significant clinical dilemma particularly in the setting of a solitary kidney or preexisting renal dysfunction. All three cases presented here involved complex renal masses, which if treated by conventional laparoscopic or open techniques, may have been associated with prolonged WITs, complications and potentially rendered the patient(s) anephric. With our described approach, the kidney is maximally protected against the insults of warm ischemia and allows for very minimal normal parenchyma excision. In fact, in our described cases, no significant major vessels were sacrificed in order to adequately dissect out the index lesions. Additionally, as a result of the meticulous dissection, very little normal renal parenchyma was excised. This was made clinically evident by stable renal function postoperatively in all patients after a median follow-up of 25.8 months.

Minimally invasive approaches to the treatment of renal masses are the gold standard and are associated with reduced postoperative pain, shorter lengths of stay, and a faster return to work [2]. Admittedly, our described approach requires longer operative times, as it is essentially the collimation of three urological procedures—laparoscopic donor nephrectomy, renorrhaphy, and renal transplantation. Some may argue that these are extraordinary lengths to preserve renal parenchyma/function, as the EORTC 30904 trial showed equivocal renal function outcomes between partial and radical nephrectomy; [5] however, this evidence applies to patients with a normal contralateral kidney. Radical nephrectomy for the index cases would have resulted in renal replacement therapy dependence of which the morbidity and complications are well established. The laparoscopic donor approach to nephrectomy may help reduce some of the morbidity associated with this technique described in earlier reports [3, 4, 6–9]. Important to note is that implicit in this procedure is the training, experience, and confidence to perform renal transplantation and vascular anastomoses. In the present series, the main urologic surgeon involved in all cases (TBM) underwent accredited, renal transplant fellowship training and is an experienced laparoscopist. Should the urologist not feel comfortable with the transplantation and vascular skills required, involvement of a vascular surgeon may be considered to facilitate this technique.

Two cases have described a laparoscopic approach to nephrectomy [10, 11] followed by* ex-vivo* partial nephrectomy for malignant renal disease, with one patient requiring post-operative hemodialysis. Despite 14 years of further technological advances and gained experience, we have shown that there still remains a role for this extraordinary technique. To put this time reference into perspective, the Da Vinci robot gained US FDA approval in 2000, with a wide array of clinical utility. Yet, as we have shown, traditional techniques still remain a critical component of effective contemporary patient care.

From an oncological standpoint, the bloodless field, provided by this technique, permits the ideal environment for meticulous tumor dissection permitting excellent oncologic control. This also facilitates preservation of vital structures and complex renorrhaphy including collecting system and segmental vessel preservation and/or reconstruction. However, it should be noted that while all three index cases were pathological T1 disease caution must be taken for tumors in the hilar region, as there is a high rate of pT3a upstaging, [12] which itself carries a significantly worse prognosis in terms for both recurrence and survival. In that situation, partial nephrectomy is likely oncologically inferior and patients would likely be better served by radical nephrectomy followed by delayed renal transplantation. In all three index cases, oncological control was maintained with up to 39 months follow-up.

In conclusion, laparoscopic nephrectomy followed by* ex vivo* partial nephrectomy and autotransplantation is a safe and viable option for the treatment of complex renal masses. This approach allows for the preservation of renal function while maintaining oncological outcomes and should be considered in the contemporary treatment paradigm of patients with complex renal masses.
